# Strategies to Control Performance of 3D-Printed, Cable-Driven Soft Polymer Actuators: From Simple Architectures to Gripper Prototype

**DOI:** 10.3390/polym10080846

**Published:** 2018-08-01

**Authors:** Viacheslav Slesarenko, Seiji Engelkemier, Pavel I. Galich, Dmitry Vladimirsky, Gregory Klein, Stephan Rudykh

**Affiliations:** 1 Faculty of Aerospace Engineering, Technion—Israel Institute of Technology, Haifa 32003, Israel; galich@campus.technion.ac.il (P.I.G.); dimka.akmid@gmail.com (D.V.); gregy.kl@tx.technion.ac.il (G.K.); 2 Department of Mechanical Engineering, Massachusetts Institute of Technology, Cambridge, MA 02139, USA; seijieng@mit.edu; 3 Department of Mechanical Engineering, University of Wisconsin-Madison, Madison, WI 53706, USA; rudykh@wisc.edu

**Keywords:** polymer actuator, soft robotics, soft machines, 3D printing, cable-driven actuation, gripper, soft composites, digital materials

## Abstract

The following is a study of the performance of soft cable-driven polymer actuators produced by multimaterial 3D printing. We demonstrate that the mechanical response of the polymer actuator with an embedded cable can be flexibly tuned through the targeted selection of actuator architecture. Various strategies, such as the addition of discrete or periodic stiff inserts, the sectioning of the actuator, or the shifting of the cable channel are employed to demonstrate ways to achieve more controllable deformed shape during weight lifting or reduce the required actuation force. To illustrate these concepts, we design and manufacture a prototype of the soft polymer gripper, which is capable of manipulating small, delicate objects. The explored strategies can be utilized in other types of soft actuators, employing, for instance, actuation by means of electroactive polymers.

## 1. Introduction

On par with conventional robots, which are made of metals and plastics and are capable of lifting heavy weights or assembling cars and planes in factories, new types of so-called “soft” polymer-based robots made of more delicate materials have gained considerable attention from scientific and engineering communities in the last two decades [1]. Currently, soft robots are not able to compete with conventional rigid robotic systems on the classical playing fields of weight lifting, fast movement, or robust response; however, they are making drastic contributions to other existing and newly emerging fields, such as medicine, food engineering, and robot-human interaction, among others [2]. Due to fundamentally new concepts, soft continuous robots and machines require a revision of the conventional actuation and control principles [2,3].

Soft robots and machines may employ a variety of actuation mechanisms, such as cable-driven actuation (CDA) [4], pneumatic actuation (PA) [5], or actuation based on electroactive polymers (EAPs) [6,7,8]. The detailed list of possible actuation mechanisms is presented in [9]; however, the three above-mentioned approaches are the most widely spread. EAPs can be divided into electronic (e.g., dielectric elastomers) and ionic EAPs (e.g., ionic polymer gels or ionic polymer-metal composites) [10]. In general, electronic EAPs require high actuation voltages, but they can produce much larger strains and respond faster compared to ionic EAPs. The most pronounced disadvantage of ionic EAPs, which outweighs their benefits, is their inability to work outside of aqueous media. However, the requirement of constant hydration is not an obstacle for soft robotic systems that operate in water, such as fish-like robots of diverse designs [8,9]. PA in soft robotics found widespread application in the creation of pneumatic artificial muscles (PAMs) and pneumatic soft manipulators. In the simple case, PAM represents a flexible, tubular structure with an empty chamber, which is filled with air in order to contract or extend a muscle [11,12]. More complex pneumatic manipulators, such as a tentacle-like soft robotic manipulator [13], consist of several sections and pressurized chambers to maintain more accurate and flexible control over actuator shape. While usually such manipulators demonstrate remarkable freedom of movement and large extension strains, they are not able to generate large forces and are prone to buckling under compression loading. Another shortcoming that restricts the applicability of PAMs is their need for compressed air. This requirement limits the sustainability of such systems and their capability to miniaturize [14]. CDA relies on external or embedded motors, whose motion is transferred to the structure via cables [4,15]. The main advantages of this mechanism are the relative simplicity of control and good scalability. CDA is characterized by low additional weight, fast response time, and long range transmission of force and power [16]. Moreover, CDA can be coupled with other actuation mechanics in order to transfer forces to machine parts, where it is challenging or ineffective to position a pneumatic chamber.

Since soft robots have infinite degrees of freedom by design, maintaining robust control over their shape is a challenging task [1]. One way to solve this control problem is to use multiple actuators, which can be triggered separately in order to achieve a desirable response from the manipulator. For instance, 15 servomotors in a cable-driven octopus arm [15] can be actuated independently, which enables precise control over the shape and movement of the tentacle. Using several pressurized chambers that can be inflated independently, one can achieve very complex, controllable deformation of pneumatic manipulators [13]. An alternative method of creating complex deformed shapes (without the need of multiple actuators) is through the smart programming of geometry and the choice of appropriate materials. With this approach, even simple actuation can lead to complex deformation patterns and movement. For instance, additional inserts of inelastomeric material around a soft silicone matrix with only a single pneumatic chamber can force a manipulator to respond to pneumatic actuation with different modes of motion, including bending, contraction, and twisting [17]. Similarly, it was shown that by controlling the fiber angle in fiber-reinforced soft actuators, one can control their movement, and it is possible to tailor the performance of the actuator for specific tasks by combining various fiber arrangements in different sections of the actuator [11,18,19,20,21].

While soft manipulators containing only one actuator are relatively straightforward in design, their manufacture usually requires multiple processes, such as molding, casting, lithography, etc. This process can be significantly simplified due to the development of multi-material 3D printing, in which complex geometry containing components made of different materials can be printed in one step [22]. While additive manufacturing does not offer a wide variety of soft elastomeric materials yet, some 3D-printable polymers with high extensibility and a low Young’s modulus are already available for commercial 3D printing [23]. Besides commercially available materials, different types of soft materials ranging from silicone to hydrogels were adopted in additive manufacturing [24]. With the development of 3D printing techniques, various designs of soft robots based on the different types of actuation were produced [25]. Employing actuation by means of shape memory polymers [26], ionic polymer-metal composites [27], or polyelectrolyte hydrogels [28], the printed systems demonstrate a wide range of possible movements from simple bending [27] to sequential folding into the desired shape [29]. Smart placement of soft and stiff parts in soft robots actuated by shape memory wires [30], or gradient design in combustion-driven robots [31], were demonstrated to be beneficial for robot operation. Therefore, targeted selection of materials and geometry not only helps to create 3D-printed composites with enhanced properties [32,33,34], but it is the key to achieving the desired performance of 3D-printed actuators [20,29,35,36,37]. In this manuscript, we employ multimaterial 3D printing to manufacture simple soft actuators, driven by an embedded cable. In order to understand how composite geometry of the actuator defines the deformed shape, we explore different geometrical and material inhomogeneities in the form of inserts, notches, and stiff tubular elements. Based on our experimental observations, we design and realize a soft 3D printed gripper, which is capable of gently holding and transporting small delicate objects. We emphasize that the main goal of this study is to provide an overview of possible ways to control their performance through smart selection of geometry and materials. The study does not represent a complete performance characterization of the soft 3D-printed actuators. These observations can be expanded for the design of soft devices with other actuation principles, such as pneumatic actuation or actuation by means of active polymeric materials.

## 2. Materials and Methods

We employed a multi-material 3D printer Objet Connex 260 (Stratasys, Eden Prairie, MN, USA) to fabricate soft actuators with different inelastomeric stiff inserts. This printer employs the Polyjet printing technique for selective deposition of photopolymer droplets directly onto a build tray. After deposition of each layer with minimal thickness of 30 µm and a pass of the roller to even the top surface, two UV lamps cure the photopolymer. Since several printing heads can be installed, up to three different photopolymers can be deposited independently during printing (one additional head is reserved for hydrophobic support). Moreover, when one uses local mixing of several photopolymers in one spot, a much wider range of materials with intermediate properties becomes available. For instance, materials used in this study belong to the so-called Tango family of materials and are formed as a mixture of soft TangoPlus (Stratasys, Eden Prairie, MN, USA) and stiff VeroWhite (Stratasys, Eden Prairie, MN, USA) materials. Depending on the mass ratio of these two base polymers, the resulting materials are characterized by different Shore indexes and Young’s moduli. We should note that during transition from one material to another, a narrow mixing zone is observed [38]; however, the width of this zone does not usually exceed 40 µm. At the same time, mechanical tests on the macro [39] and micro [38] scales indicate that the bonding between materials are stronger than the weaker base material. Therefore, while the mixing zone between materials can have a slight influence on the macroscopic mechanical properties (especially for small details), UV curing ensures a perfect adhesion between materials. No interfacial debonding was observed in the present, or previous studies.

**Figure 1:**
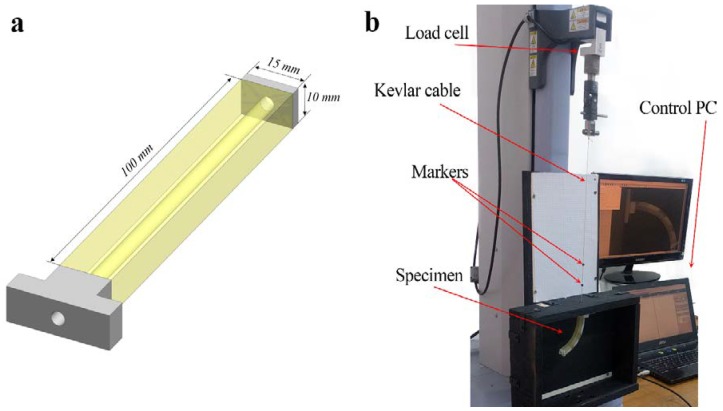
General geometry and dimensions of the actuator (**a**). The experimental setup for the testing (**b**).

To produce the actuator, we first created CAD model with required geometry in SolidWorks software (Dassault Systems, Waltham, MA, USA) (Figure 1a). In order to fix the actuator in the fixtures for mechanical testing, we added two stiff parts on both of the ends of the soft body of the actuator. After importing the CAD design to the printing software and assigning the proper materials to different parts of the specimen, we started the printing process. We note that the Polyjet technique allows us to print a whole actuator, containing several materials, in a single pass. Since jet printing does not allow unsupported elements, the empty space in the cable channel was filled with support material. After printing, the support material was scrupulously removed from the cable channel using a water jet. Soft parts of actuators were printed with soft elastomeric materials (TangoPlus, FLX9760, FLX9785), while stiff inserts and the ends of the actuators were printed in stiff VeroWhite material.

Printed actuators were mounted vertically into the fixtures, designed for the universal testing machine Shimadzu EZ-LX (Shimadzu Corporation, Kyoto, Japan), as shown in Figure 1b. Kevlar (DuPont, Wilmington, DE, USA) cable was passed through the cable channel and was connected to a small tablet at one of the ends of the specimen, while another end of the cable was tied to the top jig of the testing machine. During experiments, the top jig was moved up to retract the Kevlar cable, thus inducing deformation of the actuator. The actuator was deformed until an approximate 90 degree angle was reached, and then unloaded until zero force was measured on the load cell. The cable retraction length was measured by the vertical displacement of the top jig, while the whole actuation process was tracked by a CCD camera. In a series of experiments, we added dead weight on the free end of the actuators in order to estimate the actuator’s capacity to lift up additional weight. The force applied to the top jig and the retraction of the cable were measured during the experiments. Two small markers were placed on the cable in order to take into account the extension of the cable. However, the cable extension was negligible, since Kevlar has a very high Young’s modulus. 

## 3. Results

**Figure 2:**
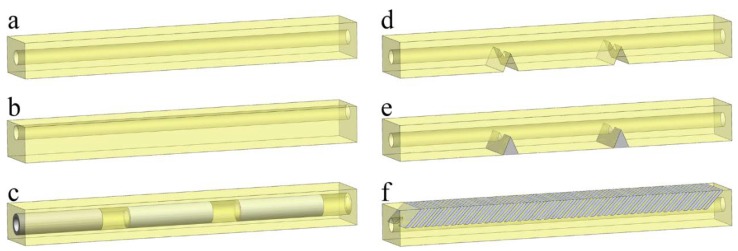
Geometry of the actuators with different channel positions and geometry. (**a**) Simple homogeneous actuator with central channel, (**b**) with shifted channel, (**c**) with stiff tubular inserts around channel, (**d**) with discrete notches, (**e**) with discrete stiff inserts, and (**f**) with periodic stiff inclusions structure. Shown colors are chosen to be similar to real colors of the 3D-printed materials. Yellow sections correspond to elastomeric material (TangoPlus, FLX9760 or FLX9785), while greyish inserts correspond to stiff VeroWhite material.

In this work, we examine the following geometrical architectures (Figure 2), because, according to our observations, they have the most significant effect on the general mechanical performance of the actuator. In particular, we examine the influence of (i) cable channel position and channel-surrounding material (a–c), (ii) discrete notches and stiff inclusions (d,e), and (iii) periodic stiff inclusions (“scales”) (f).

The considered actuators with *square* cross-sections are able to bend in two perpendicular directions simultaneously due to the symmetry of the cross-section. In order to restrict bending to one direction, we consider actuators with *rectangular* cross-sections. In this case, the flexural rigidity of the actuator in two perpendicular directions is not the same, and therefore the actuator has a well-defined bending direction. Hereafter, we solely focus on actuators with a rectangular cross-section with aspect ratio 3:2 (see Figure 1a). This particular ratio was selected in order to maintain specific bending direction, while still providing enough thickness to shift the position of the cable channel inside the actuator.

**Figure 3:**
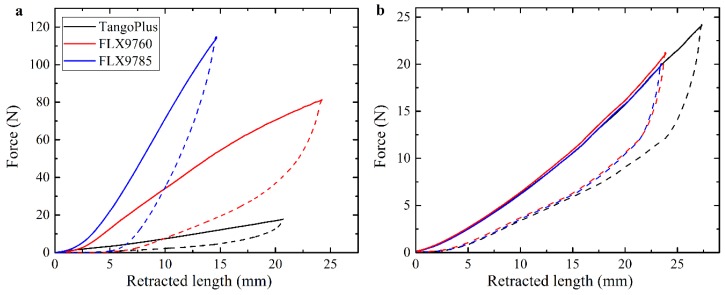
Force-retraction curves, obtained for homogeneous actuator made of different base materials (**a**). Three consecutive actuations for the homogeneous TangoPlus actuator with central position of the cable channel and 50 g dead weight attached to the free end (**b**). Solid curves represent loading, dashed curves are obtained during unloading.

As mentioned in the materials and methods sections, three different materials were used to print the main part of the soft actuators, namely, TangoPlus (E~0.6 MPa), FLX9760 (E~3 MPa), and FLX9785 (E~20 MPa) [23]. Figure 3a shows the dependencies of the force, measured by load cell, on the retracted cable length. The dependencies, shown in Figure 3a, are obtained for the homogeneous actuator with a centered cable channel (Figure 2a). Clearly, the actuators printed with stiffer materials require higher forces to achieve the same level of deformation. This aspect should be taken into account in the case of engineering applications due to limited tensile strength of the cable or limited torque of the motor. Figure 3a also reveals that loading and unloading curves do not match each other, and a hysteresis loop is observed. This effect is caused by viscoelastic properties of base materials used in 3D printing [23]. Due to the delayed response of materials, complete recovery of the initial shape does not occur immediately upon removal of loading. As the range of materials for 3D printing increases, the appearance of printable soft materials with lower damping should solve this problem. In order to check the repeatability of the actuator’s performance, each specimen was loaded and unloaded at least 3 times with one minute delay between cycles. As one can see from Figure 3b, the actuator performance remains consistent during cycling even for the actuator undergoing local bucking due to attached dead weight

### 3.1. Position of the Channel

**Figure 4:**
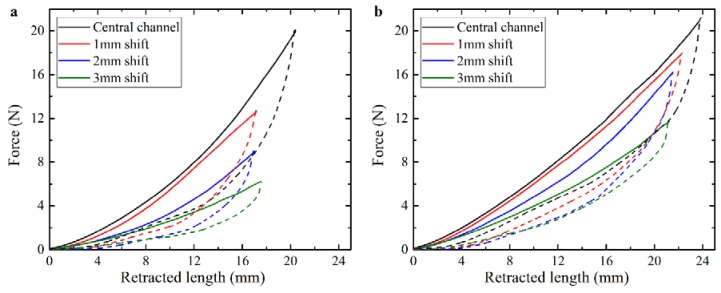
Force-retraction curves for TangoPlus actuator with different position of the channel without external load (**a**) and with additional 50 g, applied on the free end of the specimen (**b**). Solid curves represent loading; dashed curves are obtained during unloading.

**Figure 5:**
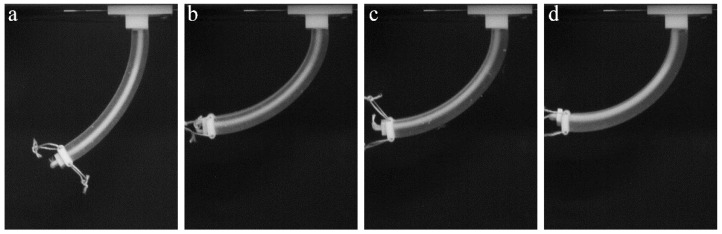
Experimental snapshots for TangoPlus actuators with central channel (**a**) and the channel shifted 1 (**b**), 2 (**c**), and 3 mm (**d**) from the central position. The deformed shape corresponds to a cable retraction length of 16 mm.

We start by considering homogeneous actuators with a straight cylindrical channel and examine the influence of moving the channel position relative to the central axis (Figure 2b). Figure 4a shows the force-retraction curves, and Figure 5 shows the corresponding snapshots, obtained during experiments on the actuators with different distances of the channel’s central axis from the center of the cross-section. It is clear that the considered actuators bend towards the direction of the channel shift. Additionally, actuators with a larger distance between the channel center and cross-section center require less force for the same cable retraction length. For instance, to achieve the deformation corresponding to a retraction of 14 mm, a force of 10 N should be applied to the actuator with the centered channel, while 4 N is enough for the actuator with the internal channel shifted 3 mm from the central position. The observed decrease in the required force is caused by the larger distance between the neutral line of the composite and the location of the applied force. From Figure 5, it is clear that the same cable retraction length does not lead to the same motions between actuators with differently located cable channels. In general, the deformation of the actuator is higher when the channel is shifted further from the central axis.

**Figure 6:**
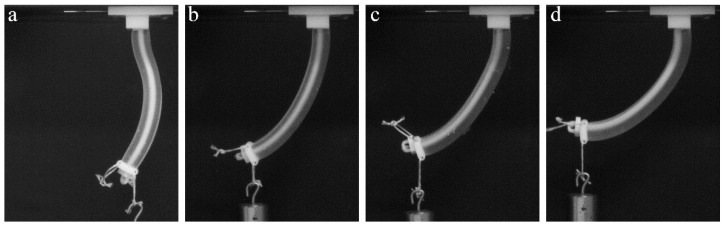
Experimental snapshots for TangoPlus actuators with central channel (**a**) and the channel shifted 1 (**b**), 2 (**c**), and 3 mm (**d**) from the central position. Additional weight is attached to the free end of the actuator in order to estimate their lifting capacity. The deformed shape corresponds to the cable retraction length of 20 mm.

Since the designed actuators hold potential engineering applications, which require lifting of weights and withstanding external loadings, we perform actuation experiments with a dead weight attached to the free end of the specimen, as shown in Figure 6. Similar to the weight-free case, actuators with a shifted channel position require a lower force in comparison to the actuator with the central cable channel (Figure 4b). While the added weight does not significantly alter the deformed shape of the actuators with a shifted channel (compare Figure 5b–d and Figure 6b–d), we observe that addition of the weight to the actuator with the central channel can lead to local buckling near the fixed end (see Figure 6a). Therefore, thoughtful placement of the cable channel inside the actuators not only reduces the tensile load on the cable but also achieves a more predictable deformation during weight lifting.

### 3.2. Actuators with Discrete Notches and Stiff Inclusions

**Figure 7:**
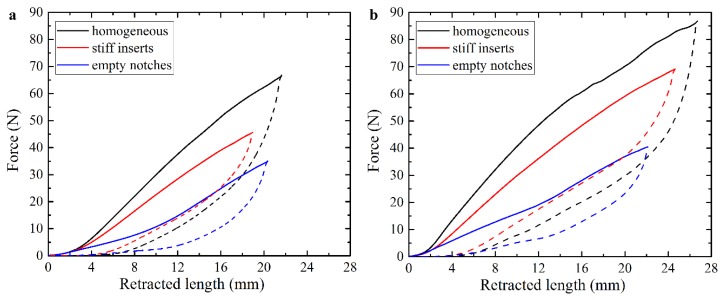
Force-retraction curves for the homogeneous FLX9760 actuator and actuators with empty notches and stiff inserts without external load (**a**) and with additional 150 g weight on the free end of the specimen (**b**).

**Figure 8:**
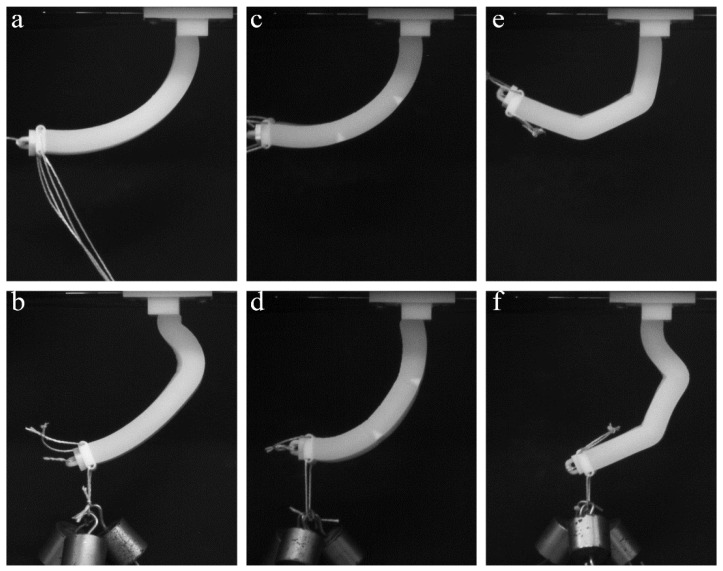
Experimental snapshots for homogeneous FLX9760 actuator (**a**,**b**), actuators with stiff inserts (**c**,**d**) and empty notches (**e**,**f**). Top row corresponds to deformed state without external loading and retraction length 19 mm. Bottom row corresponds to retraction length of 22 mm, while additional 150 g weight was applied on the free end of the actuator.

The use of non-uniform geometry, even in homogeneous materials, can be exploited to create complex deformation sequences and shapes [17]. Here, we manufacture actuators containing three sections with notches or discrete stiff inserts between sections, as shown in Figure 2d,e. These non-uniform sections in the actuator may lead to a deformed shape with non-uniform curvature in contrast to the homogeneous cases considered above. Figure 7 and Figure 8 show force-retraction curves and corresponding experimental snapshots for actuators with notches and discrete stiff inserts, along with the homogeneous actuator. The homogeneous actuator requires larger forces for the same cable retraction in comparison with the actuator containing stiff inserts, despite the higher overall Young’s modulus of the latter. At the same time, the existence of the notches drastically decreases the required force for the same cable retraction length. However, for this actuator, in contrast to the homogeneous one, the deformation mainly occurs near the notches, leading to the formation of a deformed shape with relatively sharp corners (Figure 8e). While the homogeneous FLX9760 actuator and the actuator with notches exhibit local buckling, when additional dead weight of 150 g is applied to the free end, the actuator with relatively small stiff inserts has much more uniform bending shape, which is associated with redistribution of the internal stresses due to the existence of the inserts.

### 3.3. Actuators with Periodic Stiff Inclusions

**Figure 9:**
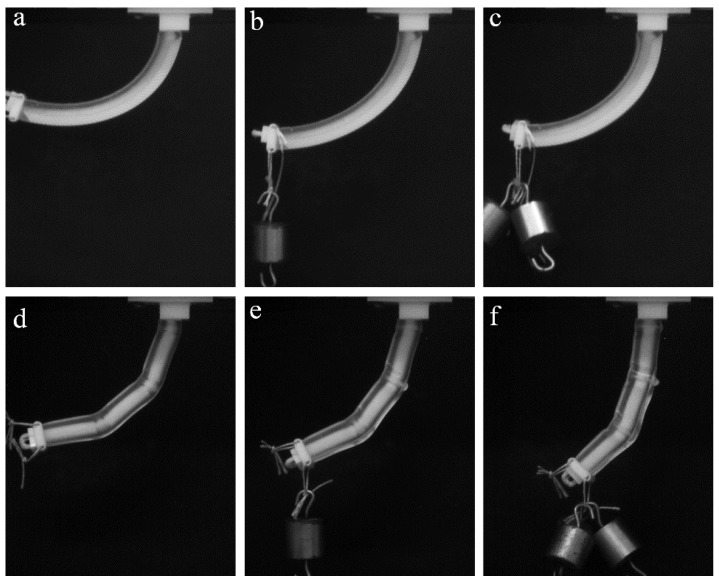
Experimental snapshot for TangoPlus actuators with periodic stiff inclusions (“scales”) (**a**–**c**) and stiff tubular sections around cable channel (**d**–**f**).

Here, we consider the mechanical behavior of a soft actuator, which contains periodic stiff inclusions on one of the sides. In contrast to the former case, in which inserts and notches are mainly exploited to separate the different sections of the specimen, the continuous pattern of the stiff inserts/inclusions introduces anisotropy through the whole length of the actuator. Here, we consider the stiff inclusions in the shape of elongated platelets, which are located on one side of the actuator. This architecture resembles an imbricated scale pattern in fish (Figure 2f) [40]. Figure 9a–c shows the snapshots, which were obtained during experiments on the “scaled” composite actuator. While the homogeneous FLX9760 actuator with a 150 g weight attached to the free end buckles near the fixed point (Figure 8b), the addition of the scales prevents such buckling, even in the actuator, which is made of much softer TangoPlus material (Figure 9a–c). Moreover, the deformed shape is characterized by uniform curvature throughout the actuator. Therefore, the addition of the periodic patterns allows one to change mechanical behavior and increase the applied maximal load without compromising the flexibility of the actuator. This observation may be important for practical applications in which actuators are required to maintain defined shapes.

### 3.4. Sectioning of the Actuator Near Cable Channel

**Figure 10:**
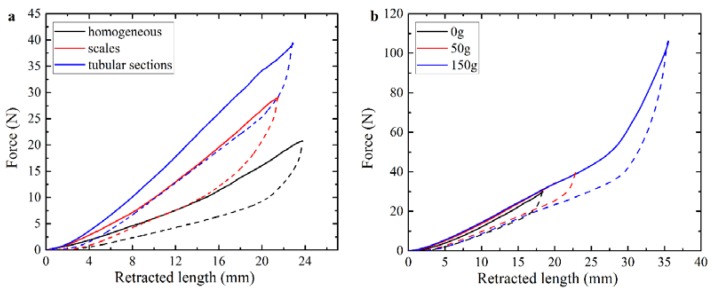
Force-retraction curves for the homogeneous TangoPlus actuator, the actuator with stiff scales, and the actuator with tubular concentric inserts around channel under additional 50 g weight on free end (**a**). Force-retraction curves for the actuator with tubular concentric inserts with different weights attached to the free end of the specimen (**b**).

Finally, we consider actuators with modifications of the channel surroundings. Remarkable mechanical performance is observed in the soft actuator with the central channel, which contains stiff cylindrical inserts around the void, splitting the actuator into three sections (Figure 2c). From the force-retraction curves (Figure 10a), it is obvious that due to the addition of the cylindrical inserts, which suppress local deformation near the channels walls, this actuator requires much higher force for deformation to occur. However, this particular structure allows us to obtain non-uniform bending motion by means of the local deformation of the soft joints between stiff tubular inclusions. We observe that such a specimen is able to demonstrate high bending deformation, which is fully reversible, in addition to being able to lift large weights (Figure 9c and Figure 10b). However, since the deformation is mainly localized inside the soft joints between the tubular inserts, these local stresses may exceed critical stress, which would lead to the failure of the actuator.

**Figure 11:**
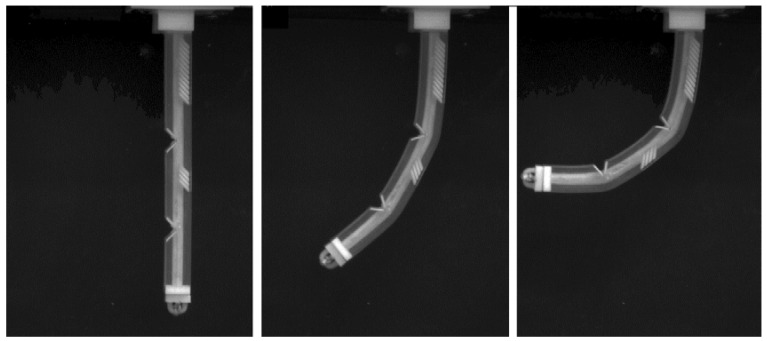
The deformation sequence of the sectioned actuator.

It is important to note that all geometrical features that are described above can be used independently, as well as in combination with each other, in order to achieve required mechanical performance. For instance, Figure 11 shows the experimental snapshots of an actuator, which contains three sections with different lengths and scale-like structure to increase the bending stiffness of each section. This design combines several features, which were considered above: empty notches program the non-uniform bending shape; stiff periodic inserts provide additional stiffness to the sections to prevent local buckling of the actuators during gripping. As one can see from Figure 11, the considered actuator remains almost straight in the top section, enforced by periodic inserts, while deformation is mostly accommodated by the empty notches. In the current design with only one cable, the deformed shape is fixed by the internal architecture of the actuator; however, a more involved design with several actuation cables can be adopted. Through independent control over these cables, a wider range of possible deformed shapes can be realized. In general, the combination of rationally designed architecture with a higher degree of freedom, available through actuation, increases ways to program more intricate shapes.

## 4. Design of the Gripper

**Figure 12:**
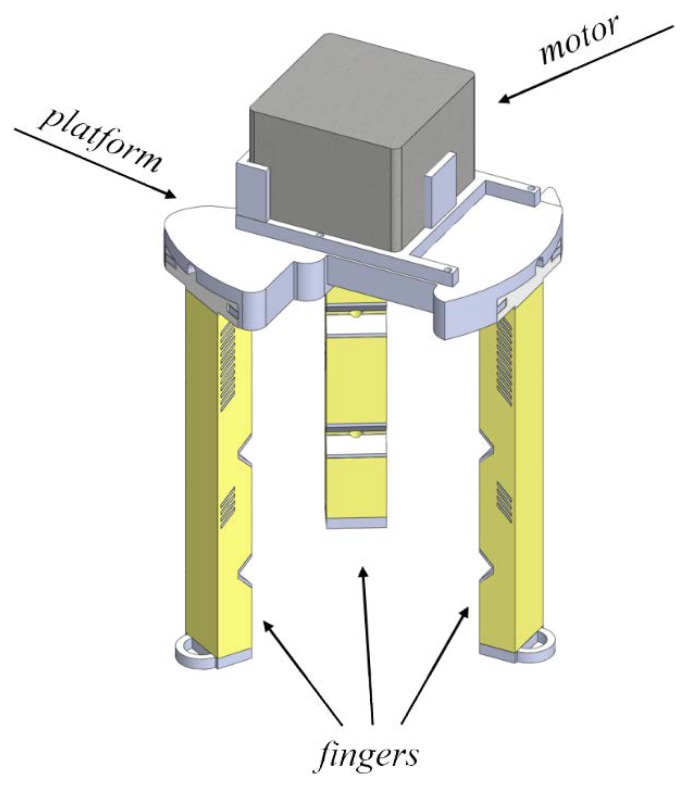
Design of the fully 3D-printed gripper with three equally spaced soft fingers and stepper motor at the top.

**Figure 13:**
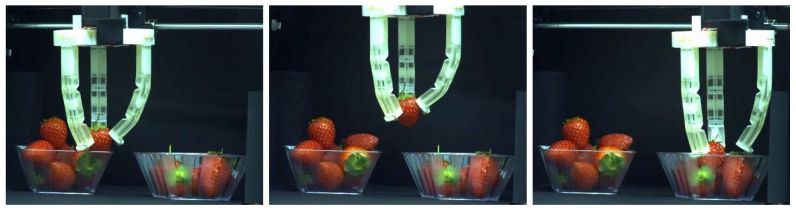
Transferring strawberries from one bowl to another using cable-actuated 3D-printed soft gripper (Video S1 in Supporting Materials).

A wide variety of robotics grippers, which are capable of manipulating small and large objects, were proposed in the existing literature [41]. They employ a variety of the actuation approaches, ranging from simple external motors [42] to electroactive polymers [43] and shape memory alloys [44]. Depending on the maturity of the underlying technology, the corresponding grippers have already reached commercialization, either in the beginning or end of their development path. However, regardless of the actuation approach, we demonstrated before, the actuator performance can be tailored by the smart choice of geometry and materials. Therefore, based on the previously shown strategy to combine a soft matrix with stiff inserts and the smart choice of geometry, we developed the gripper shown in Figure 12. This gripper consists of three sectioned actuators, shown in Figure 11, which are driven simultaneously by one Nema17 stepper motor, located on the top part. Three cables are passed through the fingers and connected to the shaft of the motor on the top, leading to the simultaneous deformation of the actuators during rotation of the motor. An alternative design with a separate motor for each finger is possible. It provides better manipulation and simultaneously increases the complexity of the control. The gripper is mounted on the platform of a conventional FDM 3D-printer, which allows us to control its vertical and horizontal movements, as well as its gripping motion through existing 3D-printing software (Repetier). Figure 13 shows the sequential process of delivering a strawberry from one bowl to another (Video S1 in Supporting Materials). This particular gripper is able to consistently lift objects with a weight of 50 g. In order to produce a gripper capable of lifting heavier objects, stiffer base materials or other architecture needs to be applied. Although this soft gripper is able to lift only relatively light objects, this design has a hidden advantage. In particular, due to the low bending stiffness of the structure, retraction of the cable does not cause a significant increase in the gripper holding force, thus providing a very gentle grip. Indeed, if the weight exceeds the potential of the gripper, local buckling near the fixed end of the actuator will occur, as shown in Figure 8b, and the gripped object will not be deformed and/or damaged. In conventional “stiff” grippers, an additional feedback loop is required in order to achieve the same behavior, whereas in soft actuators this behavior is the essential part of their mechanics.

## 5. Conclusions

We employed multimaterial 3D printing as a single-step process to manufacture soft cable-driven polymer actuators. It was shown that by using only two constituent materials, it is possible to program the performance of the soft actuator and achieve the required response through targeted choice of the geometry. By shifting the cable channel position or adding discrete and periodic stiff inserts or notches, one can control the actuation shape and increase the weightlifting capacity while maintaining a predictable shape and avoiding local buckling. The ability to combine simple cable-driven soft actuators into an assembly to transfer delicate objects demonstrates the immense potential of 3D-printed soft manipulators in real engineering applications, e.g., in food or medical industries.

## Supplementary Materials

The following are available online at http://www.mdpi.com/2073-4360/10/8/846/s1. Video S1: Manipulating raspberry by 3D-printed gripper, Video S2: Manipulating smooth ping-pong ball by 3D-printed gripper.
