# Supplementary material for: Strategies to Control Performance of 3D-Printed, Cable-Driven Soft Polymer Actuators: From Simple Architectures to Gripper Prototype
Source: Polymers (Basel). 2018 Aug 1;10(8):846. doi: 10.3390/polym10080846 (PMC6403601; doi:10.3390/polym10080846)
Supplement: Supplementary file 1 [file polymers-10-00846-s001.zip › Supplementary materials.pdf]

Supplementary materials for "Strategies to control performance of 3D-printed cable-driven soft polymer actuators: from simple architectures to gripper prototype"

V. Slesarenko[1,], S. Engelkemier[2], P. I. Galich[1], D. Vladimirsky[1], G. Klein[1], S. Rudykh[1,3]*

[1]Faculty of Aerospace Engineering, Technion – I.I.T, Israel
[2]Department of Mechanical Engineering, Massachusetts Institute of Technology, USA
[3]Department of Mechanical Engineering, University of Wisconsin–Madison, USA

We present two videos demonstrating the gripper's ability to lift up and transfer soft and delicate objects, such as strawberry (Video 1), as well as smooth spherical objects, such as a ping-pong ball (Video 2). Note that gripper alters its shape depending on the shape and weight of the object. Both videos are sped up by 2.5x, however the maximal speed of the gripper is limited mostly by the performance of motors.
